# Neuroticism increased risk of gastroesophageal reflux disease: Evidence from bidirectional Mendelian randomization

**DOI:** 10.1097/MD.0000000000050732

**Published:** 2026-09-18

**Authors:** Yan Yu, Zhifeng Fu, Jingwen Zhang, Haixing Jiang

**Affiliations:** a Department of Gastroenterology, The First Affiliated Hospital of Guangxi Medical University, Nanning, China.

**Keywords:** causal relationship, gastroesophageal reflux disease, Mendelian randomization, neuroticism

## Abstract

Gastroesophageal reflux disease (GERD) is a common gastrointestinal disorder that severely reduces quality of life. Emerging data suggest that neuroticism, a personality trait associated with increased stress reactivity and sensitivity to physiological sensations, may contribute to GERD susceptibility. However, the causality remains unknown. A bidirectional Mendelian Randomization (MR) study was conducted to evaluate the causal link between neuroticism and GERD. Genetic data for neuroticism were sourced from the UK Biobank’s genome-wide association study (GWAS) (n = 3,80,506), while GERD-associated genetic data were obtained from the FinnGen project (n = 4,17,314) for primary analysis and from the IEU Open GWAS database (n = 6,02,604) for replication and meta-analysis. Sensitivity analyses, including tests for heterogeneity and pleiotropy, were conducted to assess the robustness of causal inferences. Inverse-variance weighted (IVW) analyses revealed significant associations between certain neuroticism traits and an increased risk of GERD. The traits with the strongest associations were mood (odds ratio [OR] = 2.01, 95% confidence interval [CI] = 1.60, 2.53, *P *= 1.5 × 10^–9^), worry (OR = 1.59, 95% CI = 1.21, 2.07, *P* = .00078), and tense (OR = 1.70, 95% CI = 1.18, 2.44, *P *= .004). Weighted median and MR-Egger regression sensitivity analyses supported these findings. The results were validated in replication and meta-analysis. Reverse MR suggested GERD might influence traits like “WORRY” and “FED-UP”; however, these associations were non-significant after Bonferroni correction. Neuroticism traits, including mood, worry, and tense, may be associated with an increased risk of GERD. These findings highlight the potential role of psychological characteristics in GERD development and may provide insights into future risk assessment and management strategies for GERD.

## 1. Introduction

Gastroesophageal reflux disease (GERD) is a widespread chronic gastrointestinal disorder characterized by the reflux of stomach contents into the esophagus, commonly presenting as heartburn and regurgitation.^[1]^ GERD is quite common worldwide, with estimates indicating that 10% to 20% of people in Western populations are affected.^[2,3]^ In the United States and Europe, GERD is one of the most common gastrointestinal disorders, with the incidence increasing remarkably in recent decades.^[4]^ In addition, though historically there was a relatively lower GERD prevalence in Asia, recent research suggests an increased tendency in the prevalence of GERD, most likely due to changes in lifestyle and dietary habits.^[5,6]^

The impact of GERD is extensive, including physical consequences such as esophagitis, Barrett’s esophagus, and an increased risk of esophageal adenocarcinoma.^[7]^ Studies also reported major impairments in health-related quality of life in patients suffering from GERD.^[1]^ As reported, many people with GERD suffer from sleep disturbances, decreased work productivity, and social constraints, all of which have a negative influence on their overall well-being.^[8]^ Furthermore, GERD incurs significant healthcare costs, including expenses of periodic diagnostic tests, long-term pharmacological therapy, and, in some cases, surgical intervention.^[9]^ Thereby, the heavy socioeconomic burden of GERD emphasizes the urgency to investigate modifiable risk factors to improve preventive and management techniques.

Recent research indicates that neuroticism may facilitate the onset of GERD and exacerbate symptoms in patients with GERD.^[10,11]^ Neuroticism is a stable personality feature defined as an increased sensitivity to negative emotions, stress, and physiological sensations, and it has been confirmed to be associated with a wide range of diseases.^[12]^ Beyond its emotional reactivity, neuroticism may also involve difficulties in emotional processing and regulation, which may reflect increased psychological vulnerability. In this context, alexithymia, characterized by difficulties in identifying and describing feelings, represents a related psychological characteristic and has been associated with psychological distress and suicidal ideation in vulnerable populations.^[13]^ A majority of observational studies have found a link between neuroticism and GERD, indicating that people with high neuroticism levels experience GERD symptoms more frequently.^[14]^ Moreover, psychosocial stressors may also contribute to gastrointestinal symptoms and psychological vulnerability. The COVID-19 pandemic further highlighted the impact of psychosocial stress, with increased anxiety, psychological distress, and loneliness reported during this period, particularly among vulnerable populations.^[15]^ There might be 2 aspects of reasons to explain the association between high neuroticism levels and GERD. On the one hand, individuals with high neuroticism may be characterized by being more sensitive to visceral stimuli, more intense stress reactions, and a higher awareness of body sensations, which would consequently lead to stronger gastrointestinal stress regurgitation.^[12]^ On the other hand, annoying GERD symptoms, in turn, might also disturb the regulation of emotion, resulting in a higher level of neuroticism.^[16]^ However, observational studies typically fail to demonstrate causality, raising the possibility of reverse causation, unknown confounders, and any other undetected bias.

Mendelian randomization (MR) is an alternative approach aiming to address these problems rigorously by employing genetic variants (single nucleotide polymorphisms, SNPs) as instrumental variables (IVs), allowing causal inferences between risk factors (e.g., neuroticism) and outcomes (e.g., GERD).^[17]^ This technique effectively mitigates the confounding bias and reverse causality that are inherent in observational studies.^[18]^ Furthermore, a bidirectional MR design allows the investigation of reciprocal causality between the 2 traits, providing insight into whether neuroticism predisposes people to GERD or if GERD contributes to the development of neurotic characteristics.

In this study, we used a bidirectional MR strategy to investigate the causal relationship between neuroticism and GERD. Our goals were to determine whether neuroticism is a causative element in GERD development and whether GERD influences neuroticism. We hypothesized that genetically predicted neuroticism traits may be associated with an increased risk of GERD and that a potential bidirectional relationship may exist between neuroticism and GERD. Understanding these pathways may provide new insights into the relationship between psychological qualities and gastrointestinal problems, perhaps influencing preventive and therapeutic techniques in GERD care.

## 2. Materials and methods

### 2.1. Study design

**Figure 1.**
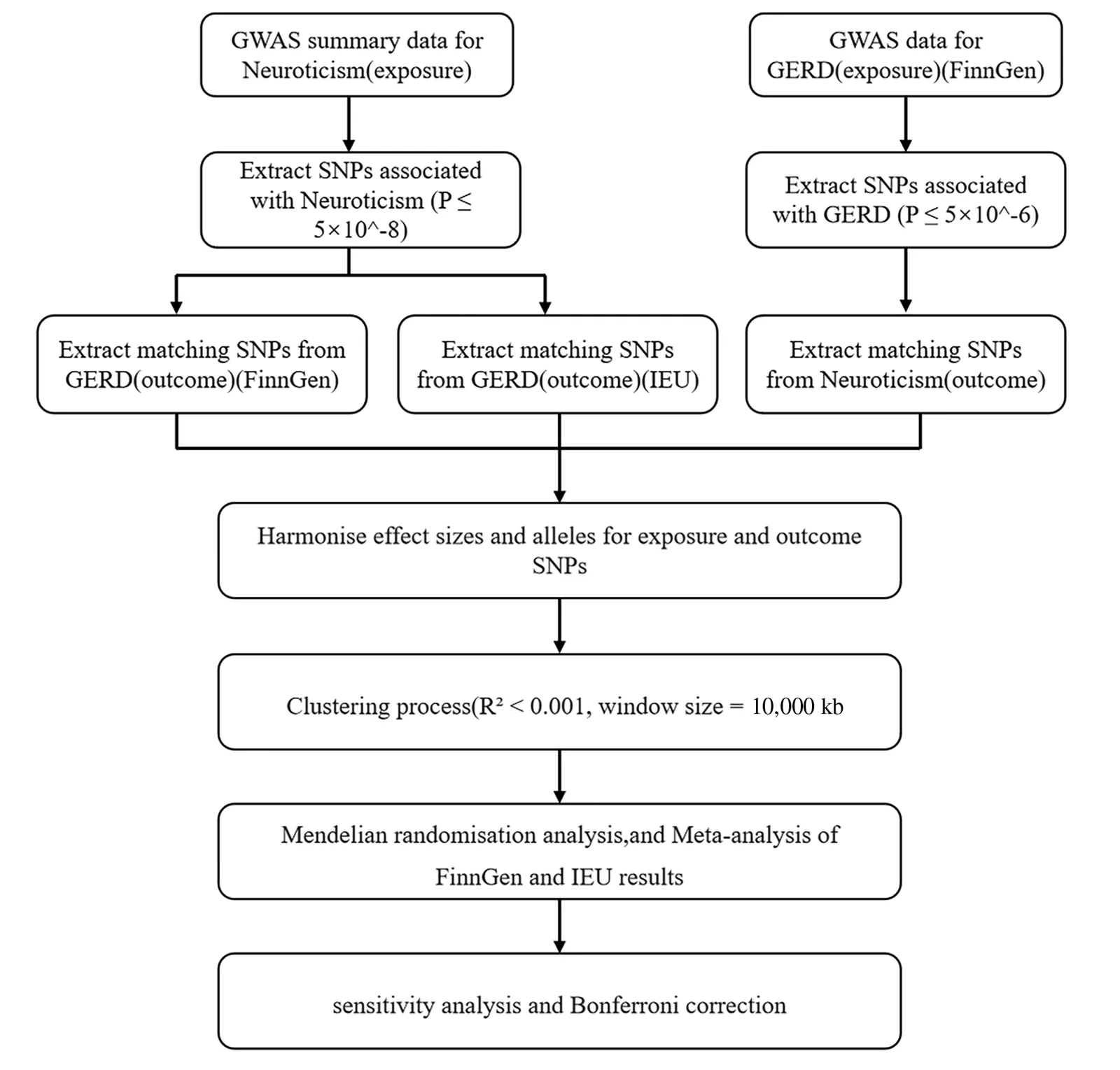
Flowchart depicting the bidirectional Mendelian randomization (MR) analysis process. GERD = gastroesophageal reflux disease, GWAS = genome-wide association studies, IEU = integrative epidemiology unit, MR = Mendelian randomization, SNP = single nucleotide polymorphism.

By utilizing genetic data from genome-wide association studies (GWAS), we investigated the causal relationship between 12 specific neuroticism items and GERD using a bidirectional MR approach. MR analysis relies on 3 core assumptions: relevance, independence, and exclusion restriction.^[19]^ The research design of the MR study is illustrated in Figure 1. This study used only publicly available GWAS summary statistics and did not involve the collection of individual-level participant data. Therefore, no additional ethical approval or informed consent was required for the present study.

### 2.2. GWAS data for neuroticism

We used summary data for item-level neuroticisms from the GWAS study conducted by Nagel et al, which examined neuroticism in a sample of 3,80,506 Europeans from the United Kingdom Biobank (UKB).^[20]^ This study found 255 distinct genomic loci associated with neuroticism, and we employed SNPs from these loci as genetic instruments in an MR analysis to look into potential causal links between neuroticism and GERD. Specifically, Nagel et al examined 12 specific neuroticism traits, including “IRR,” “LONE,” “MIS,” “MOOD,” “FED-UP,” “NERV-FEEL,” “WORRY,” “TENSE,” “SUF-NERV,” “HURT,” “WORR-EMB,” and “GUILT,” indicating that neuroticism is made up of distinct genetic components rather than a single, unified trait. A detailed definition for each of the neuroticism traits is presented in Table S1, Supplemental Digital Content 1.

### 2.3. GWAS data for GERD

Two sets of GERD GWAS data were included in this study. Specifically, we obtained genetic data for GERD from the R11 data of the FinnGen consortium, including 32,232 cases and 3,85,082 controls. The FinnGen study is a developing project that provides genetic data for thousands of endpoints. The GERD cases were diagnosed according to the International Classification of Diseases, Ninth Revision (ICD-9) or Tenth Revision (ICD-10). We also leveraged the genetic data of GERD derived from the GWAS conducted by Ong et al, consisting of 1,29,080 cases and 4,73,524 controls.^[21]^ Specifically, the data are deposited in the Integrative Epidemiology Unit (IEU) Open GWAS database and could be retrieved and extracted using the GWAS ID “ebi-a-GCST90000514.”

### 2.4. Instrument selection

Genetic instruments for each neuroticism trait were selected using predefined criteria.^[22-25]^ SNPs associated with neuroticism at genome-wide significance (*P* < 5 × 10^−8^) were extracted and clumped based on linkage disequilibrium (LD) parameters (*r*^2^ < 0.001 within 10,000 kb).^[26]^ SNPs with F-statistics < 10 were excluded to minimize weak instrument bias.^[19]^ Exposure-associated SNPs were then harmonized with outcome datasets, excluding SNPs associated with outcomes, missing SNPs, palindromic SNPs, and incompatible alleles. Only neuroticism traits with more than 2 available SNPs were included in MR analyses.^[27]^

### 2.5. Primary analysis

For primary analysis, we used the GWAS data for GERD from the FinnGen program to avoid sample overlapping with the exposure dataset. In this work, the inverse-variance weighted (IVW) method was the primary analytical technique for evaluating causal effects. IVW combines ratio estimates from each variant, weighted by the inverse of their variance, to improve statistical power.^[28]^ The significance criterion for the IVW estimates was set at *P *< .0042 using a Bonferroni correction (0.05/12), which helped control for type I error.^[29]^

### 2.6. Sensitivity analyses

To assess the robustness of our findings, supplementary analyses were conducted using the weighted median method.^[30]^ In addition, MR-Egger regression was performed as a complementary analytical approach.^[31]^ We also conducted several sensitivity analyses to validate the MR findings. Cochran *Q* test was used to assess heterogeneity. When heterogeneity was detected (*P* < .05, *I*^2^ > 25%), Radial MR was used to identify and exclude outliers, followed by outlier-corrected IVW analysis to verify the robustness of the MR estimates.^[32]^ Horizontal pleiotropy was assessed using the MR-Egger intercept, with a non-significant intercept indicating limited pleiotropic bias (*P* > .05).^[31]^ Leave-one-out analysis was conducted to evaluate the influence of individual SNPs on the pooled IVW estimates.^[33]^

By integrating the results of these analyses, neuroticism traits were considered to be involved in GERD development if they met the following criteria: consistent direction and magnitude across the IVW, MR-Egger, and weighted median methods; no evidence of pleiotropy; no heterogeneity, or consistent IVW estimates after removal of outliers identified by Radial MR; and no influential SNPs identified in the leave-one-out analysis.

### 2.7. Replication and meta-analysis

To verify the results obtained from the primary analysis, replicative IVW analysis was conducted using the GERD GWAS data from the study conducted by Ong et al.^[21]^ Besides, the results from primary analysis and replicative analysis were then meta-analyzed to yield final causal estimates.

### 2.8. Reverse analysis

We also conducted a reverse analysis to evaluate the effect of GERD on neuroticisms. Analytical steps were as mentioned above. Notably, given no sufficient SNPs available at *P* < 5 × 10^-8^ for GERD, we relaxed the significance threshold to 5 × 10^-6^.

### 2.9. Statistical software

All analyses were performed in the R environment (version 4.4.1) with the “TwoSampleMR” package (version 0.6.8). Additionally, the Review Manager (RevMan) software (version 5.4.1) was used for meta-analysis.

## 3. Results

### 3.1. Neuroticism and GERD risk

After strict IV selection, the number of SNPs for all 12 neuroticism items ranged from 7 to 40, with all *F*-statistics over 10, showing strong instrumental validity (Table S2, Supplemental Digital Content 2). The harmonized data were presented in Table S3, Supplemental Digital Content 3.

### 3.2. Primary analysis

**Figure 2.**
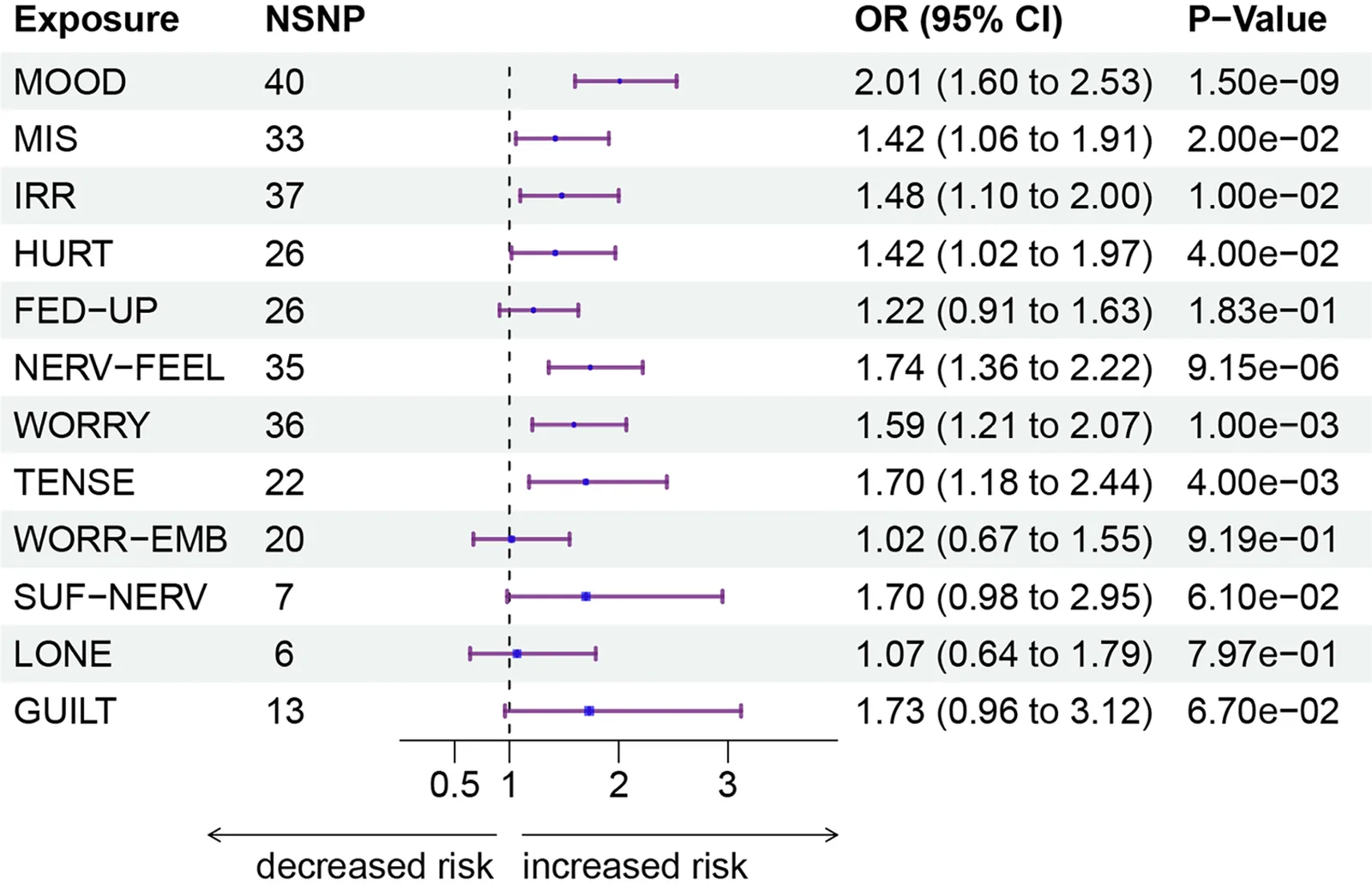
Forest plot for the causal effect of specific neuroticism dimensions on the risk of gastroesophageal reflux disease (GERD) derived from inverse-variance weighted (IVW) analysis. CI = confidence interval, GERD = gastroesophageal reflux disease, IVW = inverse-variance weighted, NSNP = number of single nucleotide polymorphisms, OR = odds ratio.

**Figure 3.**
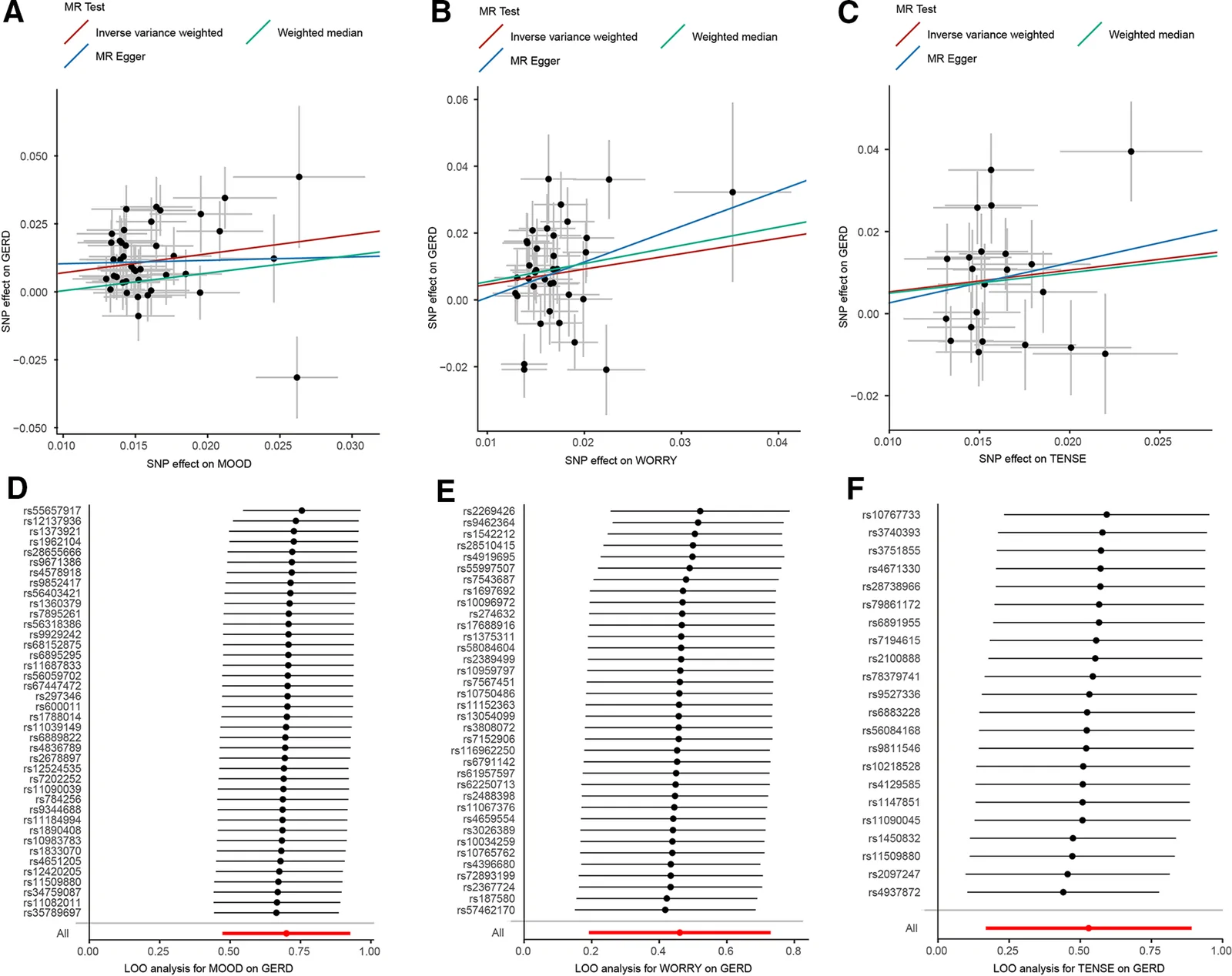
Scatterplot for the significant Mendelian randomization (MR) association (*P* < .05) between neuroticism and gastroesophageal reflux disease (GERD). (A), (B), and (C) represent the scatterplots for specific neuroticism and GERD, while (D), (E), and (F) represent the corresponding leave-one-out analysis. Forest plots for the MR leave-one-out analysis of the significant inverse-variance weighted (IVW) estimates. Within each panel, the black points represent the causal estimate of the association between neuroticism and GERD after discarding each SNP in turn. Red points represent the pooled IVW estimates. Horizontal lines denote 95% confidence intervals. GERD = gastroesophageal reflux disease, IVW = inverse-variance weighted, MR = Mendelian randomization, SNP = single nucleotide polymorphism.

The IVW analysis found substantial relationships between specific neuroticism dimensions and GERD (Fig. 2). After Bonferroni correction, we found several neuroticism items significantly associated with an increased risk of GERD. The trait “MOOD” was most strongly associated with GERD, with odds ratio (OR) = 2.01, 95% confidence interval (CI) = 1.60, 2.53, *P* = 1.50 × 10^–9^. The “NERV-FEEL” characteristic showed a significant correlation (OR = 1.74, 95% CI = 1.36, 2.22, *P *= 9.15 × 10^−6^). Other significant relationships were “WORRY” (OR = 1.59, 95% CI = 1.21, 2.07, *P *= .00078) and “TENSE” (OR = 1.70, 95% CI = 1.18, 2.44, *P *= .004). For the neuroticism items “MOOD,” “WORRY,” and “TENSE,” the weighted median and MR-Egger methods showed similar estimates with the IVW estimates (Fig. 3). Besides, the MR-Egger intercept test presented non-significant intercepts, suggesting no horizontal pleiotropy existed (Table 1). While Cochran *Q* test revealed heterogeneity (Table 1), Radial MR analysis was used to detect and remove influential outliers. Re-analysis with IVW yielded consistent results, and leave-one-out analysis confirmed that no single SNP had a dominant influence on the relationships (Fig. 3).

**Table 1:** Sensitivity analysis for the causal association between neuroticism and GERD.

Exposure	Outcome	Nsnp	WM		Egger		Heterogeneity		Pleiotropy	
			OR (95% CI)	*P*	OR (95% CI)	*P*	*Q (I*^2^)	*P*	Intercept	*P*
MOOD	GERD	40	1.71 (1.28, 2.29)	3.36 × 10^-4^	1.13 (0.26, 4.91)	.868	56.39	.035	0.017	.404
MIS	GERD	33	1.47 (1.04, 2.06)	0.028	0.74 (0.23, 2.41)	.616	63.12	.001	0.011	.269
IRR	GERD	37	1.32 (0.96, 1.81)	0.087	0.57 (0.06, 5.77)	.634	86.51	4.83 × 10^-6^	−0.010	.490
HURT	GERD	26	1.51 (1.04, 2.19)	0.030	0.43 (0.10, 1.94)	.284	50.74	.002	−0.007	.740
FED-UP	GERD	26	1.16 (0.83, 1.63)	0.377	1.35 (0.29, 6.28)	.702	41.40	.021	0.019	.126
NERV-FEEL	GERD	35	1.86 (1.39, 2.50)	3.70 × 10^-5^	0.58 (0.16, 2.16)	.423	50.50	.034	−0.002	.891
WORRY	GERD	36	1.72 (1.28, 2.33)	3.73 × 10^-4^	2.90 (0.52, 16.16)	.233	65.78	.001	0.009	.442
TENSE	GERD	22	1.64 (1.10, 2.47)	0.016	2.65 (0.19, 36.15)	.474	44.09	.002	0.015	.418
WORR-EMB	GERD	20	1.05 (0.67, 1.62)	0.843	0.37 (0.03, 3.97)	.421	47.70	2.83 × 10^-4^	0.018	.106
SUF-NERV	GERD	7	1.51 (0.85, 2.69)	0.163	0.71 (0.02, 30.30)	.865	10.51	.105	−0.038	.205
LONE	GERD	6	0.92 (0.49, 1.73)	0.800	13.52 (0.49, 376.18)	.200	5.63	.344	0.015	.664
GUILT	GERD	13	1.34 (0.81, 2.23)	0.251	0.18 (0.003, 10.80)	.432	39.81	7.74 × 10^-5^	0.037	.298

CI = confidence interval, GERD = gastroesophageal reflux disease, Nsnp = number of single nucleotide polymorphisms, OR = odds ratio, WM = weighted median.

We also found several neuroticism traits showed suggestive associations with GERD, including “MIS” (OR = 1.42, 95% CI = 1.06, 1.91, *P *= .02), “IRR” (OR = 1.48, 95% CI = 1.10, 2.00, *P *= .01), and “HURT” (OR = 1.42, 95% CI = 1.02, 1.97, *P *= .04). However, dimensions such as “FED-UP,” “WORR-EMB,” “SUF-NERV,” “LONE,” and “GUILT” presented no evidence of associations with GERD (*P* > .05). Mendelian random estimation of the causal relationship between neuroticism and GERD, results of sensitivity analyses, and leave-one-out analyses are described in detail in Tables S4 and S5, Supplemental Digital Content 4.

### 3.3. Replication and meta-analysis

**Figure 4.**
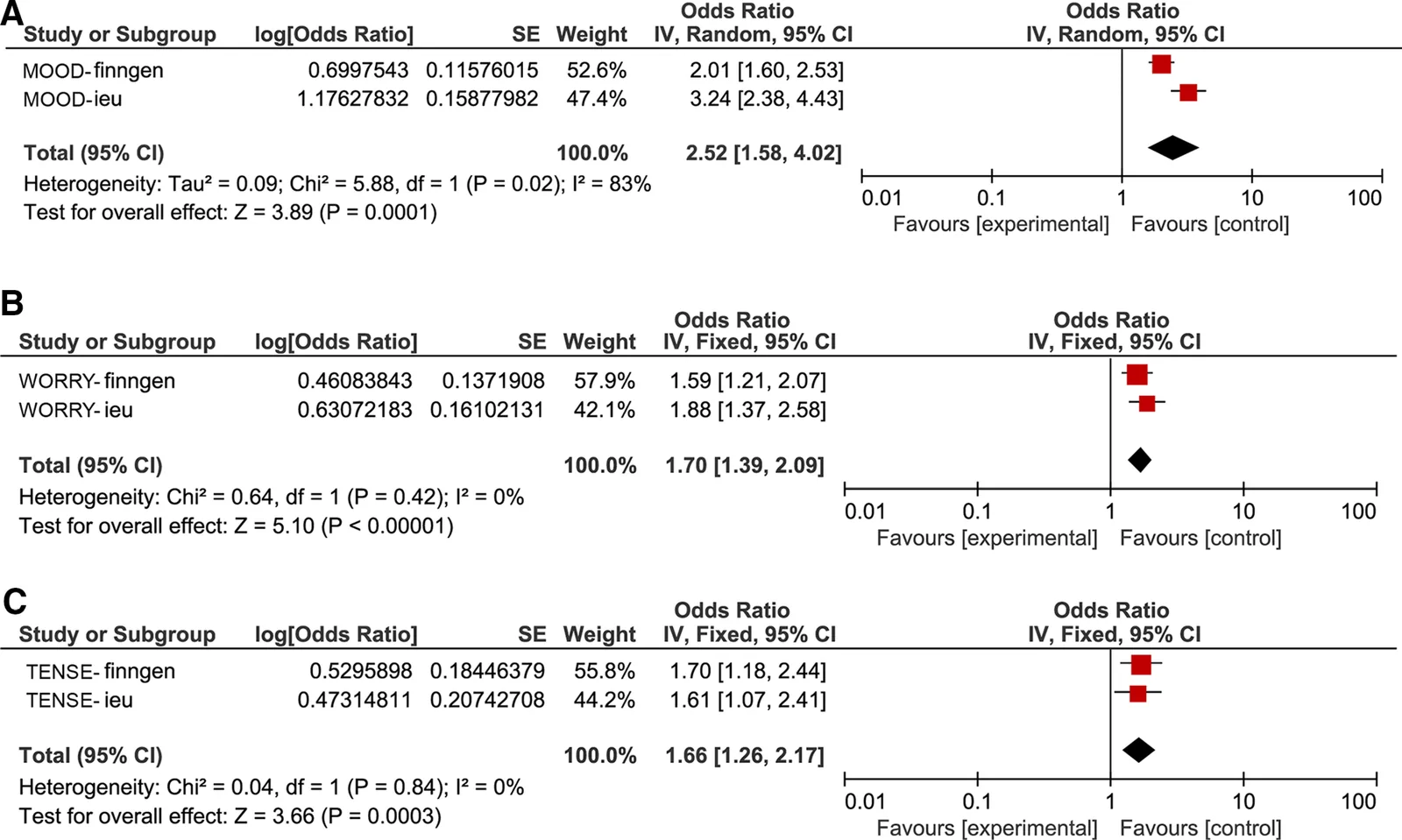
Meta-analysis of the causal associations between neuroticism traits and gastroesophageal reflux disease (GERD). CI = confidence interval, SE = standard error, GERD = gastroesophageal reflux disease, IV = inverse variance.

Replication in an independent GERD cohort from the IEU Open GWAS database confirmed the relationship between neuroticism traits and GERD (Fig. 4). A meta-analysis of FinnGen and IEU data found a pooled OR of 2.52 (95% CI = 1.58, 4.02) for “MOOD” using a random-effects model, despite significant heterogeneity (*I*^2^ = 83%). The meta-analysis for “WORRY” revealed an OR of 1.70 (95% CI = 1.39, 2.09) with no heterogeneity (*I*^2^ = 0%). Similarly, “TENSE” provided an OR of 1.66 (95% CI = 1.26, 2.17) with no heterogeneity (*I*^2^ = 0%).

### 3.4. GERD and neuroticism traits

After Bonferroni correction, there were no significant associations between GERD and neuroticism traits. The IVW analysis detected that genetic liability to GERD was nominally associated with several neuroticism traits (*P* < .05), including “FED-UP” (OR = 1.031, 95% CI = 1.006, 1.058, *P* = .017), “WORRY” (OR = 1.032, 95% CI = 1.008, 1.056, *P* = .01), “WORR-EMB” (OR = 1.038, 95% CI = 1.006, 1.070, *P* = .019), “LONE” (OR = 1.030, 95% CI = 1.004, 1.056, *P* = .022) and “GUILT” (OR = 1.03, 95% CI = 1.007, 1.061, *P* = .013). The weighted median and MR-Egger regression corroborated the associations for “FED-UP” and “WORRY” (Table 2), implying that GERD might potentially influence these characteristics. “WORRY” demonstrated no heterogeneity or pleiotropy (Table 2). The MR-Egger intercepts for “FED-UP” were not significant, indicating modest pleiotropic bias (Table 2), while the Cochran *Q* test revealed some heterogeneity, the Radial MR analysis assisted in reducing outliers, and IVW re-analysis supported the findings. Additionally, leave-one-out analysis found that no single SNP had a significant effect on these associations. Detailed in Tables S6 and S7, Supplemental Digital Content 5.

**Table 2:** Mendelian randomization estimates and sensitivity analysis results for GERD and neuroticism traits.

Exposure	Outcome	Nsnp	IVW		WM		Egger		Heterogeneity		Pleiotropy	
			OR (95% CI)	*P*	OR (95% CI)	*P*	OR (95% CI)	*P*	Q (I2)	*P*	Intercept	*P*
GERD	MOOD	29	1.014 (0.988, 1.040)	.304	1.015 (0.985, 1.046)	.325	1.020 (0.965, 1.079)	.488	43.210	.033	−0.0004	.794
GERD	MIS	29	1.012 (0.990, 1.034)	.280	1.010 (0.981, 1.041)	.501	1.009 (0.963, 1.057)	.715	30.206	.353	0.0002	.890
GERD	IRR	29	1.013 (0.988, 1.038)	.315	1.012 (0.982, 1.042)	.446	1.028 (0.973, 1.085)	.334	40.073	.065	−0.0009	.561
GERD	HURT	29	1.008 (0.981, 1.036)	.552	1.005 (0.974, 1.038)	.738	0.994 (0.937, 1.055)	.844	48.825	.009	0.0009	.598
GERD	FED-UP	29	1.031 (1.006, 1.058)	.017	1.035 (1.001, 1.070)	.041	1.048 (0.992, 1.107)	.103	41.895	.044	−0.0011	.514
GERD	NERV-FEEL	29	1.012 (0.991, 1.033)	.264	1.012 (0.983, 1.042)	.416	1.046 (1.001, 1.094)	.058	27.764	.477	−0.0022	.110
GERD	WORRY	29	1.032 (1.008, 1.056)	.010	1.032 (1.003, 1.063)	.031	1.053 (1.001, 1.108)	.054	36.131	.139	−0.0014	.369
GERD	TENSE	29	1.024 (0.996, 1.052)	.093	1.017 (0.984, 1.051)	.312	1.058 (0.998, 1.122)	.069	49.325	.008	−0.0022	.223
GERD	WORR-EMB	29	1.038 (1.006, 1.070)	.019	1.016 (0.980, 1.053)	.393	1.000 (0.936, 1.068)	.991	61.790	.0002	0.0024	.219
GERD	SUF-NERV	29	1.017 (0.994, 1.040)	.153	1.013 (0.983, 1.045)	.401	1.058 (1.010, 1.109)	.024	33.316	.224	−0.0026	.066
GERD	LONE	29	1.030 (1.004, 1.056)	.022	1.021 (0.990, 1.054)	.188	1.033 (0.978, 1.092)	.255	41.947	.044	−0.0002	.892
GERD	GUILT	29	1.033 (1.007, 1.061)	.013	1.024 (0.993, 1.055)	.130	1.025 (0.969, 1.085)	.396	44.471	.025	0.0005	.765

CI = confidence interval, GERD = gastroesophageal reflux disease, Nsnp = number of single nucleotide polymorphisms, OR = odds ratio, WM = weighted median.

## 4. Discussion

Using the MR technique, this work found that 3 neuroticism traits were associated with a higher risk of GERD, including MOOD, WORRY, and TENSE. In the other direction, this MR work did not reveal a significant effect of GERD on neuroticism. To the best of our knowledge, this is the first MR study to systematically evaluate the association between distinct dimensions of neuroticism and GERD.

Previous research has studied how psychological elements such as emotional instability, stress, and anxiety connect to GERD. Emotional instability is commonly comorbid with exacerbated GERD symptoms, probably attributed to excessive gastric acid output and insufficient lower esophageal sphincter (LES) function, both of which can exacerbate acid reflux^[16,34,35]^. Stress, in particular, has been proven to increase acid sensitivity and mucosal permeability, making the esophagus more susceptible to injury.^[36]^ In addition, stress and anxiety-related activities such as overeating, eating GERD-triggering foods, and smoking might aggravate GERD symptoms.^[37,38]^

In this bidirectional Mendelian randomization analysis, we evaluated the causal relationship between neuroticism and GERD, finding significant associations between specific neurotic traits – particularly MOOD, WORRY, and TENSE – and an increased risk of GERD. The mood trait, which encompasses aspects like emotional instability, frequent mood swings, feelings of misery, and general discontent, showed the strongest association with GERD. Worry, characterized by a tendency toward excessive concern or rumination about past or future events, and tense, which relates to physical or psychological tension and a predisposition to stress, followed closely.^[20]^ These results are consistent with observational research linking neurotic characteristics to gastrointestinal problems. This work improves causal inference by employing a Mendelian randomization strategy to reduce confounding and handle potential reverse causation.

Several potential mechanisms may account for the association between neuroticism and GERD. Elevated stress reactivity, which is common in people with high neuroticism, activates the hypothalamic-pituitary-adrenal (HPA) axis and raises cortisol levels, which can consequently exert a deleterious impact on gastrointestinal function.^[39]^ Furthermore, increased visceral sensitivity in neurotic people makes them focus more on GERD symptoms, presumably due to enhanced pain signals in the central nervous system.^[40]^ Altered behavioral patterns might also be a possible mechanism linking neuroticism to GERD. For instance, increased worry and maladaptive coping mechanisms may increase the risk of GERD by encouraging habits that exacerbate GERD symptoms, such as irregular eating and smoking.^[41]^ Recognizing these mechanisms would help facilitate a comprehensive strategy to manage GERD through addressing underlying psychological factors.

Although initial results revealed that GERD might influence certain neurotic qualities, such as “WORRY” and feeling “FED-UP,” these correlations failed to pass Bonferroni correction. Nonetheless, prolonged GERD discomfort may exacerbate neurotic tendencies by activating a feedback loop that affects mental health via increased stress sensitivity and social disengagement.^[16]^ More studies are warranted to further validate this potential.

Our findings indicate that neuroticism may be an important factor involved in the development of GERD, which highlights that neuroticism may serve as a promising target for GERD prevention and treatment. By focusing on neuroticism-related behaviors and responses, it may be possible to reduce GERD risk or symptom intensity. Psychological therapies, such as cognitive-behavioral therapy (CBT) and stress management practices, may offer individuals tools to better manage emotional responses, potentially reducing stress-related effects on the digestive system. Such strategies may increase resilience and lessen the risk of GERD symptoms developing as a result of daily stress. As a result, including mental health support into primary care for people with high neuroticism could give a more comprehensive approach to GERD prevention, addressing both mental and physical health concerns.

This study’s strengths include its bidirectional MR design, the use of large-scale GWAS data, and rigorous sensitivity analyses. The consistency of the results across several MR methods, as well as replication in external independent datasets, further supports the validity of our findings. To enhance the robustness of the causal estimates, GERD genetic data were obtained from 2 large independent datasets, including the FinnGen collaboration and the IEU Open GWAS database, and a meta-analysis was performed to integrate these datasets. Multiple sensitivity analyses, including weighted median analysis, MR-Egger regression, Cochran *Q* test, radial MR analysis, and leave-one-out analysis, were conducted to evaluate potential pleiotropy, heterogeneity, and result stability. These methodological approaches further strengthen the reliability of our findings.

Several limitations should be acknowledged. First, although multiple sensitivity analyses were conducted to evaluate the robustness of our findings, the possibility of residual horizontal pleiotropy cannot be completely excluded in MR analyses. Second, the GWAS datasets included predominantly individuals of European ancestry, which may limit the generalizability of our findings to other populations. Third, neuroticism is a complex psychological construct, and the genetic instruments used in this study may not fully represent all dimensions of neuroticism-related traits. Finally, further studies involving diverse populations and prospective clinical and functional investigations are needed to validate our findings and determine whether interventions targeting neuroticism-related characteristics could influence the risk or clinical outcomes of GERD.

## 5. Conclusion

Our findings provide genetic evidence supporting potential causal associations between specific neuroticism-related traits and the risk of GERD, particularly “MOOD,” “WORRY,” and “TENSE.” These findings highlight the potential role of psychological characteristics in the development of GERD and may contribute to future risk assessment and management strategies for GERD. They also provide new insights into the complex interactions between psychological and gastrointestinal disorders and contribute to a better understanding of GERD pathogenesis. Further studies involving diverse populations and prospective clinical investigations are warranted to validate these findings and to determine whether interventions targeting neuroticism-related characteristics can improve GERD prevention and clinical outcomes.

## Acknowledgments

We would like to express our gratitude to all the participants and researchers of the UK Biobank, the FinnGen consortium, and the IEU OpenGWAS database for providing valuable GWAS data. Their contributions have significantly facilitated our exploration of the causal relationship between neuroticism and gastroesophageal reflux disease. Additionally, we would like to thank all those who contributed to data organization, analysis, and study design.

## Author contributions

**Conceptualization:** Yan Yu, Haixing Jiang.

**Data curation:** Yan Yu.

**Formal analysis:** Yan Yu.

**Investigation:** Yan Yu.

**Methodology:** Yan Yu.

**Supervision:** Zhifeng Fu, Haixing Jiang.

**Validation:** Zhifeng Fu.

**Visualization:** Jingwen Zhang.

**Writing – original draft:** Yan Yu.

**Writing – review & editing:** Jingwen Zhang, Haixing Jiang.














